# Mutations of *CYP1B1 and FOXC1* genes for childhood glaucoma in Japanese individuals

**DOI:** 10.1007/s10384-024-01103-0

**Published:** 2024-08-19

**Authors:** Nobuo Fuse, Masae Kimura, Ai Shimizu, Seizo Koshiba, Teruhiko Hamanaka, Makoto Nakamura, Nobuo Ishida, Hiroshi Sakai, Yoko Ikeda, Kazuhiko Mori, Atsushi Endo, Masao Nagasaki, Fumiki Katsuoka, Jun Yasuda, Yoichi Matsubara, Toru Nakazawa, Masayuki Yamamoto

**Affiliations:** 1 Department of Integrative Genomics, Tohoku Medical Megabank Organization, 2-1 Seiryo- machi, Aoba-ku, Sendai, 980-8573 Miyagi Japan; 2 Department of Ophthalmology, Tohoku University Graduate School of Medicine, 1-1 Seiryo- machi, Aoba-ku, Sendai, 980-8574 Miyagi Japan; 3 Department of Ophthalmology, Japanese Red Cross Medical Center, 4-1-22 Hiroo, Shibuyaku, 150-8935 Tokyo, Japan; 4 Division of Ophthalmology, Department of Surgery, Kobe University Graduate School of Medicine, 7-5-2 Kusunoki-cho, Chuo-ku, 650-0017 Kobe, Japan; 5 Ishida Eye Clinic, 2-2-31 Honcho, Joetsu-shi, Niigata, 943-0832 Japan; 6 Urasoe Sakai Eye Clinic, 6-1-21 Miyagi, Urasoe, 901-2126 Okinawa Japan; 7 Department of Ophthalmology, Kyoto Prefectural University of Medicine, 465 Kajiicho, Kawaramachi Hirokouji, Kamigyo-ku, Kyoto, 602-0841 Japan; 8 Altech Corporation, Muscat Building 6F, 3-7-13, Nagamachi, Taihaku-ku, Sendai, 982-0011 Miyagi Japan; 9 Human Biosciences Unit for the Top Global Course Center for the Promotion of Interdisciplinary Education and Research (CPIER), Kyoto University, 53, Shogoinkawahara-cho, Sakyo-ku, Kyoto City, 606-8507 Kyoto Japan; 10 National Center for Child Health and Development, Research Institute 2-10-1 Okura Setagaya-ku, Tokyo, 157-8535 Japan

**Keywords:** Childhood glaucoma, Primary congenital glaucoma, *CYP1B1* gene, *FOXC1* gene, Whole-exome sequencing (WES)

## Abstract

**Purpose:**

To explore the frequency and positions of genetic mutations in *CYP1B1* and *FOXC1* in a Japanese population.

**Study design:**

Molecular genetic analysis.

**Methods:**

Genomic DNA was extracted from 31 Japanese patients with childhood glaucoma (CG) from 29 families. We examined the *CYP1B*,* FOXC1*, and *MYOC* genes using Sanger sequencing and whole-exome sequencing (WES).

**Results:**

For *CYP1B1*, we identified 9 families that harbored novel mutations, p.A202T, p.D274E, p.Q340*, and p.V420G; the remaining mutations had been previously reported. When mapped to the CYP1B1 protein structure, all mutations appeared to influence the enzymatic activity of CYP1B1 by provoking structural deformity. Five patients were homozygotes or compound heterozygotes, supporting the recessive inheritance of the *CYP1B1* mutations in CG. In contrast, four patients were heterozygous for the *CYP1B1* mutation, suggesting the presence of regulatory region mutations or strong modifiers. For the *FOXC1* gene, we identified 3 novel mutations, p.Q23fs, p.Q70R, and p.E163*, all of which were identified in a heterozygous state. No mutation was found in the *MYOC* gene in these CG patients. All individuals with *CYP1B1* and *FOXC1* mutations were severely affected by early-onset CG. In the *CYP1B1-*, *FOXC1-*, and *MYOC-*negative families, we also searched for variants in the other candidate genes reported for CG through WES, but could not find any mutations in these genes.

**Conclusions:**

Our analyses of 29 CG families revealed 9 families with point mutations in the *CYP1B1* gene, and four of those patients appeared to be heterozygotes, suggesting the presence of complex pathogenic mechanisms. *FOXC1* appears to be another major causal gene of CG, indicating that panel sequencing of *CYP1B1* and *FOXC1* will be useful for diagnosis of CG in Japanese individuals.

**Supplementary Information:**

The online version contains supplementary material available at 10.1007/s10384-024-01103-0.

## Introduction

Childhood glaucoma (CG) comprises a group of disorders characterized by elevated intraocular pressure (IOP) caused by an abnormality of the aqueous humor outflow system with subsequent optic nerve damage and devastating vision impairment. CG is divided into two subtypes: primary glaucoma without systemic abnormalities and secondary glaucoma with systemic abnormalities. CG comprises a heterogeneous group of disorders and occurs in children younger than 4 years of age. CG is characterized by enlargement of the globe (buphthalmos), edema, opacification of the cornea with rupture of Descemet’s membrane (Haab’s striae), and progressive glaucomatous optic atrophy. Primary congenital glaucoma (PCG) occurs in approximately 1 in 20,000 live births in Western countries [1, 2], while the incidence of PCG is as high as 1:2,500 live births among Saudi Arabians [3]. The highest incidence reported is 1:1,250 in Slovakian Gypsies [4], suggesting a genetic background in the pathogenesis of PCG.

It has been proposed that PCG is an autosomal recessive developmental disorder [4, 5]. Several genes and/or loci are reported to be causative and are referred to as GLC (glaucoma) loci; for example, *CYP1B1* [6] is located at the GLC3A locus (2p21) [7], and *LTBP2* is located at the GLC3D locus (14q24.3) [8]. These loci exhibit recessive inheritance. In contrast, *TIE2* (*TEK*) [9] and *ANGPT1* [10] have been shown to be autosomal dominant genes for PCG.

Of these causative genes and loci for PCG, the *CYP1B1* gene (OMIM 601771) is the major contributor to CG [3, 11]. The *CYP1B1* gene encodes a subtype of cytochrome P450 monooxygenase that metabolizes endogenous compounds, including 17b-estradiol, retinoic acid, arachidonic acid, and melatonin [12, 13]. A targeted knockout study of the *CYP1B1* gene in homozygous mice reveals developmental abnormalities partially mimicking those of CG [14], further supporting the notion that the *CYP1B1* gene is a causative gene of PCG. The *CYP1B1* gene has also been examined in Japanese CG patients, and mutations in this gene have been found to cover approximately 20% of Japanese CG patients [15–17].

Secondary CG accompanies anterior segment dysgenesis of the eye and has a genetically heterogeneous spectrum [18]. For instance, Axenfeld–Rieger anomaly (ARA), a developmental anomaly classified as secondary CG, is caused by a mutation in the *Forkhead Box C1* (*FOXC1*) gene (OMIM 601090) [19]. The *FOXC1* gene encodes a member of the winged helix/forkhead family of transcription factors. Mutations in the *FOXC1* gene are also found in patients with a spectrum of allelic anterior segment disorders, such as iridogoniodysgenesis anomaly, associated with secondary CG transmitted with an autosomal dominant pattern [20]. These findings imply a potential role for *FOXC1* in the development of ocular tissues, including drainage structures. Heterozygous missense and frameshift mutations in the *FOXC1* gene have been found in 5 (2.38%) out of 210 CG patients [21] and 8 (4.8%) out of 166 patients with a suspected diagnosis of CG [22]. While many CG-related genes, such as *LTBP2* [8], *TEK* [9], *ANGPT1* [10], *PITX2*, *PXDN* [23, 24] and *CPAMD8* [25], are reported to be involved, these mutations are rare. In a large childhood and early-onset glaucoma registry, biallelic variants in *CYP1B1* and heterozygous variants in *FOXC1* and *MYOC* were most commonly reported among probands [26]. In this study, we decided to concentrate on the variants in *CYP1B1*,* FOXC1*, and *MYOC*.

While mutations in the *CYP1B1*,* FOXC1* and *MYOC* genes appear to be involved in the pathogenesis of CG, the precise relationships between point mutations in these genes and clinical phenotypes remain to be determined. Thus, gene mutation–clinical phenotype relationships in CG should be validated much more profoundly through in-depth clinical genome analyses. To gain further insights, we decided to further characterize the CG patients. In this study, we screened for genetic mutations in 29 families and 31 cases of CG treated by our team. The results showed highly frequent involvement of the *CYP1B1* and *FOXC1* gene mutations in CG patients (31% and 10%, respectively), but the results also revealed intricate inheritance patterns of mutations in the *CYP1B1* gene leading to primary CG development.

## Patients and methods

In this study, we targeted families thought to have recessive inheritance or sporadic cases. Routine ophthalmic examinations were performed. We studied 31 Japanese patients with CG younger than 3 years of age from 29 families, which became prevalent before 3 years of age (22 men and 9 women) and 9 unaffected family members. Each family had a pedigree pattern with suspected recessive inheritance or a sporadic pattern. Parental consanguinity was not present in any of the patients. All patients were subjected to eye examinations, including an evaluation of their clinical features via slit lamp biomicroscopy, ophthalmoscopy, tonography, and gonioscopy. The patients with CG included patients initially diagnosed with PCG who presented elevated IOP associated with corneal edema, rupture of Descemet’s membrane, or buphthalmos before 3 years of age. The purpose and procedures of the study were explained to all adult patients, and informed consent was obtained from the legal guardians of the children. In addition, we obtained informed assent when possible. This study was approved by the Institutional Review Board of each institute. This study was conducted in accordance with the Declaration of Helsinki, the Ethical Guidelines for Human Genome/Gene Analysis Research, and other appropriate guidelines.

**Fig. 1:**
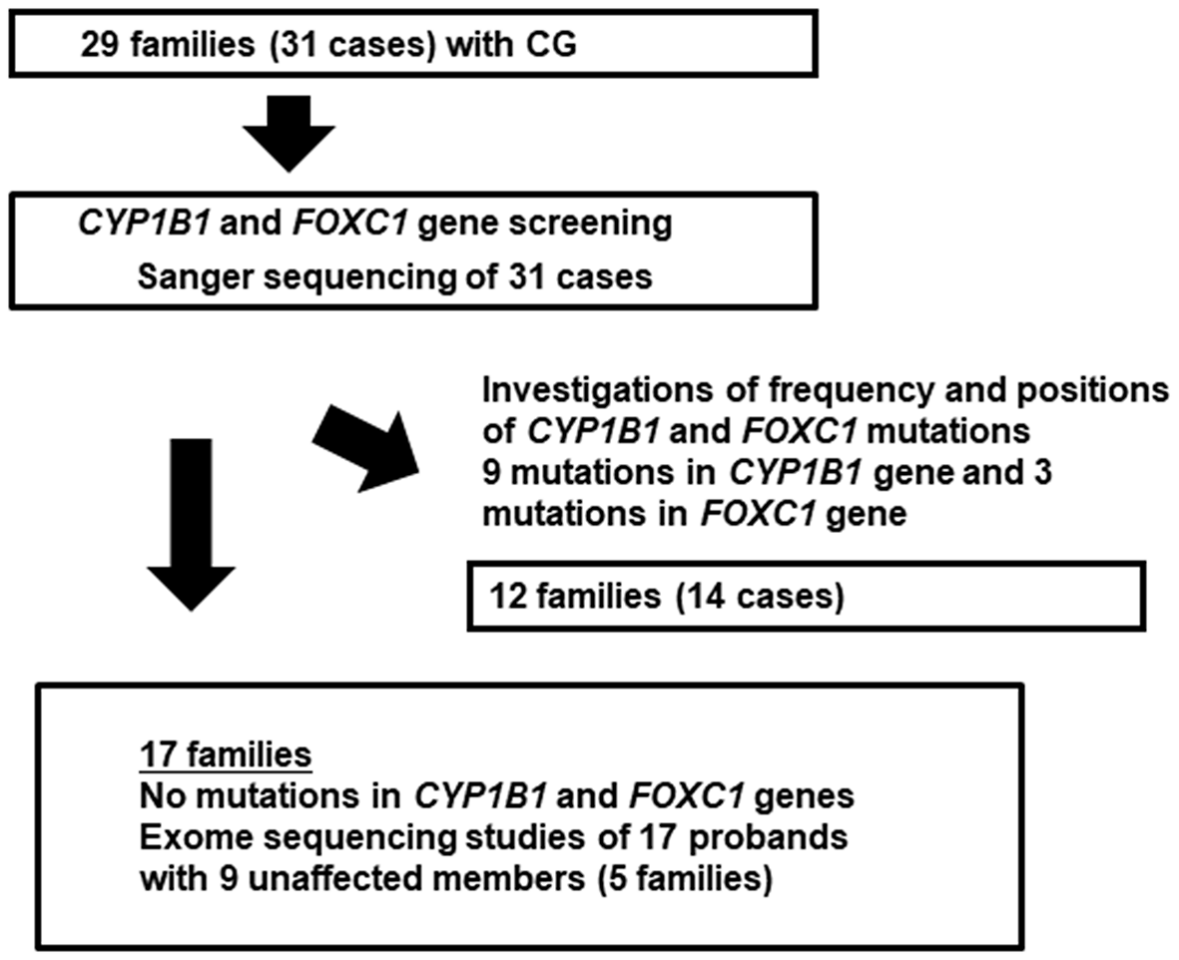
Experimental design of this study. In the first screen, we performed CYP1B1 and FOXC1 gene screenings for 31 CG patients in 29 families. The results showed that 9 families were positive for CYP1B1 gene mutations, 3 families were positive for FOXC1 gene mutations, and 17 families had no mutation in either the CYP1B1 or FOXC1 gene

The design of the experiments conducted in this study is shown in Fig. 1. Genomic DNA was extracted from leukocytes from peripheral blood and purified with a Qiagen QIAamp Blood Kit (Qiagen). Sanger sequencing of the *CYP1B1* and *FOXC1* genes was performed by determining the DNA sequences of PCR-amplified regions of the genes from both affected and unaffected individuals. The PCR primers used to amplify the DNA fragments encoding amino acid residues are shown in Supplementary Table S1. PCR was performed in an amplification mixture (50 µL) containing 200 ng of template genomic DNA, primers at 0.5 µM, and 1 U of Ex *Taq* polymerase (Takara Bio) for *CYP1B1* screening or 1 U of KOD FxNeo (Toyobo) for *FOXC1* screening. The *CYP1B1* gene was amplified via initial denaturation at 95 °C for 5 min; 30 cycles of denaturation at 95 °C for 30 s, annealing at 62 ~ 65 °C (depending on the primer set) for 30 s, and extension at 72 °C for 30 s; and the *FOXC1* gene was amplified via initial denaturation at 94 °C for 2 min followed by 30 cycles of 98 °C for 10 s and extension at 68 °C for 1 min using KOD FxNeo polymerase. The purified fragments were directly sequenced using a BigDye Terminator Cycle Sequencing Ready Reaction Kit (Applied Biosystems) on an automated DNA sequencer (Model 3500 or 3730 Genetic Analyzer: Thermo Fisher Scientific). The *MYOC* mutations in the coding regions were also assessed with direct sequencing in the same manner as previously reported [27]. To assess allele frequencies, we used the 38 K Whole-genome Japanese SNP Databases from the Tohoku Medical Megabank Project [28, 29] and Genome Aggregation Database (gnomAD) total allele frequencies.

According to the 38 K Japanese Whole-Genome Reference Panel [28, 29], approximately 1% of patients are carriers of a probable single pathogenic *CYP1B1* mutation. In the United States, the expected carrier frequency is 0.89% [30]. In this study we selected variants that had an allele frequency of less than 0.01. Variants were also annotated using the Clin Var and Human Gene Mutation Database (HGMD) Professional^®^ to search for disease-causing mutations. Pathogenic mutations in the Clin Var or DM (disease-causing mutation) according to the HGMD Professional were defined as mutations. When Clin Var and HGMD Professional evaluation could not be obtained, we referred to the standards and guidelines for the interpretation of sequence variants of the American College of Medical Genetics and Genomics (ACMG) [31] for evidence of pathogenicity. Mapping of the protein structure of *CYP1B1* mutants was carried out in heterozygous CG patients to confirm structural deformities in the CYP1B1 protein.

As we could not identify variants in the *CYP1B1*, *FOXC1* or *MYOC* genes in the remaining 17 CG patients, we conducted whole-exome sequencing of the 17 probands and 9 unaffected members in 5 families utilizing a HiSeq 2500 sequencer and a standard protocol [28].

## Results

### Targeted sequencing of the *CYP1B1* gene

We recruited families that had a pedigree pattern with suspected recessive inheritance or a sporadic pattern. As summarized in Fig. 1, we performed targeted Sanger sequencing of the *CYP1B1* and *FOXC1* genes for the 31 CG patients and identified mutations in the *CYP1B1* gene in 9 patients and mutations in the *FOXC1* gene in 3 patients but no mutations in the *MYOC* gene in these CG patients. Among the patients with *CYP1B1* gene variants, four new variants were identified in this study, p.A202T, p.D274E, p.Q340* and p.V420G. On the other hand, five mutations, p.W57*(* indicates stop codon) [32, 33], p.S215I [34], p.I324fs [16], p.V364M [16] and p.D430E [17], have been reported previously and HGMD Professional indicates these four variants as DM (disease-causing mutation) (Table 1). For the four new variants i.e., p.A202T, p.D274E, p.Q340* and p.V420G, we applied standards and guidelines for the interpretation of sequence variants of ACMG. Variants p.A202T and p. D274E are at least applicable to strong pathogenic criteria (PS: pathogenic strong), and 3 moderate pathogenic criteria (PM: pathogenic moderate). Variant p.Q340* is applicable to very strong pathogenic criteria (PVS: pathogenic very strong) and 1 PS. Variant p.V420G is applicable to 1 PS, 2PM, and 2 supporting pathogenic criteria (PP: pathogenic supporting), and we finally classified four variants as a pathogenic (Table 1). In the *FOXC1* gene, all 3 variants, i.e., p.Q23fs, p.Q70R and p.E163*, are newly identified in this study. Variants p.Q23fs and p.E163* are applicable to very strong pathogenic criteria (PVS) and 1 strong pathogenic criteria (PS). Variant p.Q70R is applicable to 1 strong pathogenic criterion (PS) and 3 moderate pathogenic criteria (PM) of ACMG interpretation. Clinical evaluation of subjects with *CYP1B1* mutations revealed that 80% of the patients were bilaterally affected with a severe disease course, and almost all patients experienced early onset of the disease, i.e., at less than 3 months of age. In addition, the early onset of CG in patients with *FOXC1* mutations was similar to that in patients with *CYP1B1* mutations.

**Table 1:** *CYP1B1* gene mutations found in this study

Family no.	Subject no.	Exon	AA change	Base change^1^	SNP	Gene location of mutations (Domain)	Allele frequency in ToMMo 38KJPN ^2^	Allele frequency in gnomAD browser^3^ (total)	CADD score	Clin var	HGMD variant class	Disease phenotype in HGMD	ACMG interpretation
119	G1152	2	p.W57*	c.G171A	rs72549387	-	0.00015	0.000401	24.9	Pathogenic	DM	Peters’ anomaly	-
053	G987	2	p.A202T	c.G604A	rs1488240339	E-helix	0	0.000001	30	NA	NA	NA	PS4,PM1,2,3,PP3
108	G558	2	p.S215I	c.G644T	rs72549384	-	0.000568	0.000048	22	US	DM	PCG	-
129	G1199	2	p.D274E	c.C822G	NA	G-helix	0	NA	8.9	NA	NA	NA	PS4,PM1,2,3,PP4
053	G987	2	p.I324fs	c.972_973 insAT	NA	I-helix	0	NA	-	NA	DM	PCG	-
129	G1199	2	p.Q340*	c.C1018T	NA	I-helix	0	NA	40	NA	NA	NA	PVS1, PS4, PM1,2
006,118, 119,132	G551,552,1150, 1152, 1207	3	p.V364M	c.G1090A	rs72549379	J-helix	0.000981	0.000020	22.3	Pathogenic	DM	PCG	-
020	G493	3	p.V420G	c.T1259G	NA	β1-3-sheet	0	0.000001	26.6	NA	NA	NA	PS4,PM1,2,PP3,4
040	G279	3	p.D430E	c.C1290G	rs201181935	-	0.000775	0.000022	9.2	US	DM	PCG	-

Reference homo/hetero/alternative homo

1. Reference sequence NM_000104.4.　2. The allele frequency of reference alleles was derived from the 38KJPN database distributed by the Tohoku Medical Megabank Organization. 3. gnomAD (Genome Aggregation Database) v4.0.0

NA; Not applicable, US; Uncertain significance, DM; Disease-causing mutation, PCG; Primary gongenital glaucoma, D: Probably damaging, PD: Possibly damaging, B: Benign, PS: Pathogenic strong, PM: Pathogenic moderate, PP: Pathogenic Supporting, PVS: Pathogenic very strong

In the *CYP1B1-*,* FOXC1-*,* and MYOC-*negative families, we also searched carefully for variants in the other candidate genes reported for CG, including *LTBP2*,* TEK*,* ANGPT1*,* PITX2*,* PXDN* and *CPAMD8*, through whole-exome sequencing, but we could not find any mutations in these genes.

### Relationships between clinical manifestations of CG and mutations in the *CYP1B1* gene

**Fig. 2:**
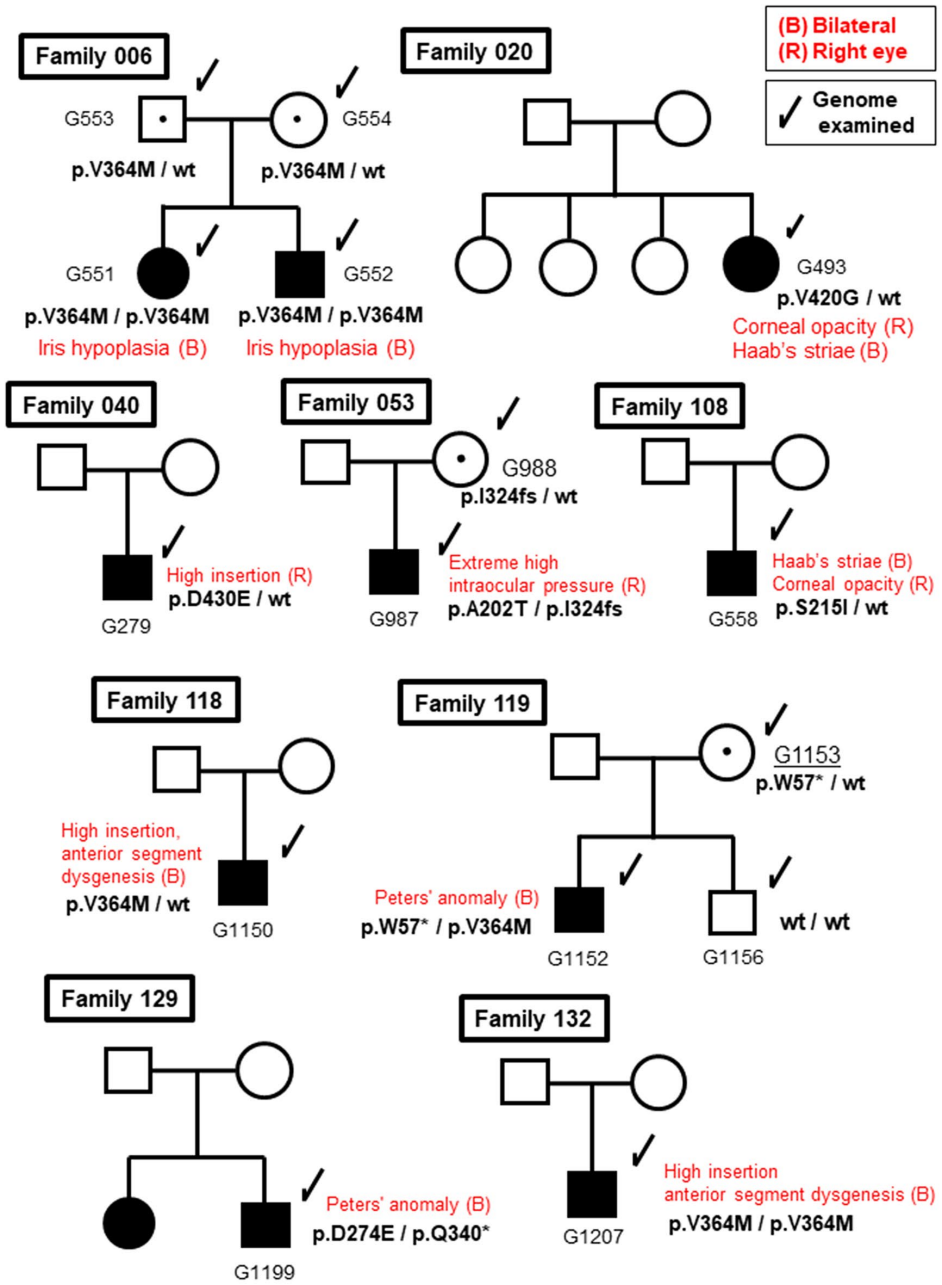
Pedigrees of the families, including members with CG with *CYP1B1* mutations. The genotypes are indicated beside the symbols. Arrows indicate family members whose genomes were examined. Closed boxes and circles indicate subjects with CG. Mutation carriers are indicated by black dots in the center. Open boxes and circles indicate subjects with no CG phenotypes. CG: childhood glaucoma. wt: wild-type allele

We examined the pedigree of patients with *CYP1B1* gene mutations. As shown in Fig. 2, two families were found to be homozygous for p.V364M/p.V364M variant (Family 006 and Family 132). Family 006 included a homozygous sister and brother, along with a heterozygous (carrier) father and mother. Three families presented compound heterozygous mutations: p.A202T/p. I324fs (Family 053), p.W57*/p.V364M (Family 119), and p.D274E/p.Q340* (Family 129). Notably, Family 119, which harbored the p.W57*/p.V364M mutations exhibited Peter’s anomaly with anterior segment dysgenesis, high insertion of the iris, peripheral anterior synechiae, and corneal opacity. In addition, Family 129, which harbored the p.D274E/p.Q340* mutations, also exhibited Peter’s anomaly with anterior segment dysgenesis, corectopia, and high insertion of the iris. In contrast, members of Family 006, which harbors the p.V364M heterozygote mutation, showed no signs of CG or anterior segment dysgenesis.

All *CYP1B1* mutation patients exhibited early onset (infantile onset > 1–24 months), and 80% of the patients were bilaterally affected (Table 2). All patients received trabeculotomy or goniotomy as the first intervention, and six out of ten patients underwent trabeculectomy or a tube shunt operation as an additional procedure.

**Table 2:** *CYP1B1* gene: genotype–phenotype correlations with mutations reported in this study

Family & Subject No.	Sex	AA change	Age at onset	Age at gene testing	Affected eye	Surgical intervention		Current IOP (mmHg) *R*/L	Visual acuity *R*/L	Clinical phenotype
						*R*	L			
006 G551	F	p.V364 M/p.V364M	FMB	14	Bilateral	2xTLO	1xTLO	22/18	1.5/1.5	B) Iris stromal hypoplasia with loss of crypts
006 G552	M	p.V364 M/p.V364M	FMB	10	Bilateral	1xTLO	1xTLO	20/18	1.5/1.5	B) Iris stromal hypoplasia with loss of crypts
020 G493	F	p.V420G/ND	FMB	50	Bilateral	8x TLO, goniotomy	2x TLO, 2x TLE	28/12	NLP/0.1	R) Corneal opacity, B) Haab’s striae
040 G279	M	p.D430E/ND	2 y.o.	20	Right	Goniotomy, TLE	NONE	12/12	0.15/1.5	R) High insertion
053 G987	M	p.A202T/p.I324fs	FMB	8	Right	1x TLO	NONE	NA	1.5/1.5	R) Extreme high IOP
108 G558	M	p.S215I/ND	FMB	55	Bilateral	1x TLO	1x TLO, 2x TLE	6/18	NLP/HM	B) Corneal enlargement (> 13 mm), Haab’s striae, R) Corneal opacity
118 G1150	M	p.V364 M/ND	FMB	20	Bilateral	Multiple procedure	Multiple procedure, 1X tube shunt	NA/14	NLP/HM	B) Anterior segment dysgenesis, high insertion of the iris
119 G1152	M	p.W57*/p.V364M	FMB	18	Bilateral	Multiple procedure	Multiple procedure 2X tube shunt	NA/18	NLP/0.01	B) Peters’ anomaly, anterior segment dysgenesis, corneal opacity, high insertion of the iris
129 G1199	M	p.D274E/p.Q340*	1 y.o.	24	Bilateral	2x TLO, 2x TLE	2x TLO, 2x TLE	9/15	0.8/0.2	B) Peters’ anomaly, anterior segment dysgenesis, corectopia, high insertion of the iris
132 G1207	M	p.V364 M/p.V364M	FMB	1	Bilateral	1x TLO	2x TLO	22/22	LP/0.4	B) High insertion, anterior segment dysgenesis, corneal opacity

M: Male, F: Female, FMB: First few months after birth, TLO: Trabeculotomy, TLE: Trabeculectomy, NA: Not applicable, NLP: No light perception, HM: Hand movement; LP: Light perception

### Mapping of *CYP1B1* mutations in CG patients on genome and protein structure data

**Fig. 3:**
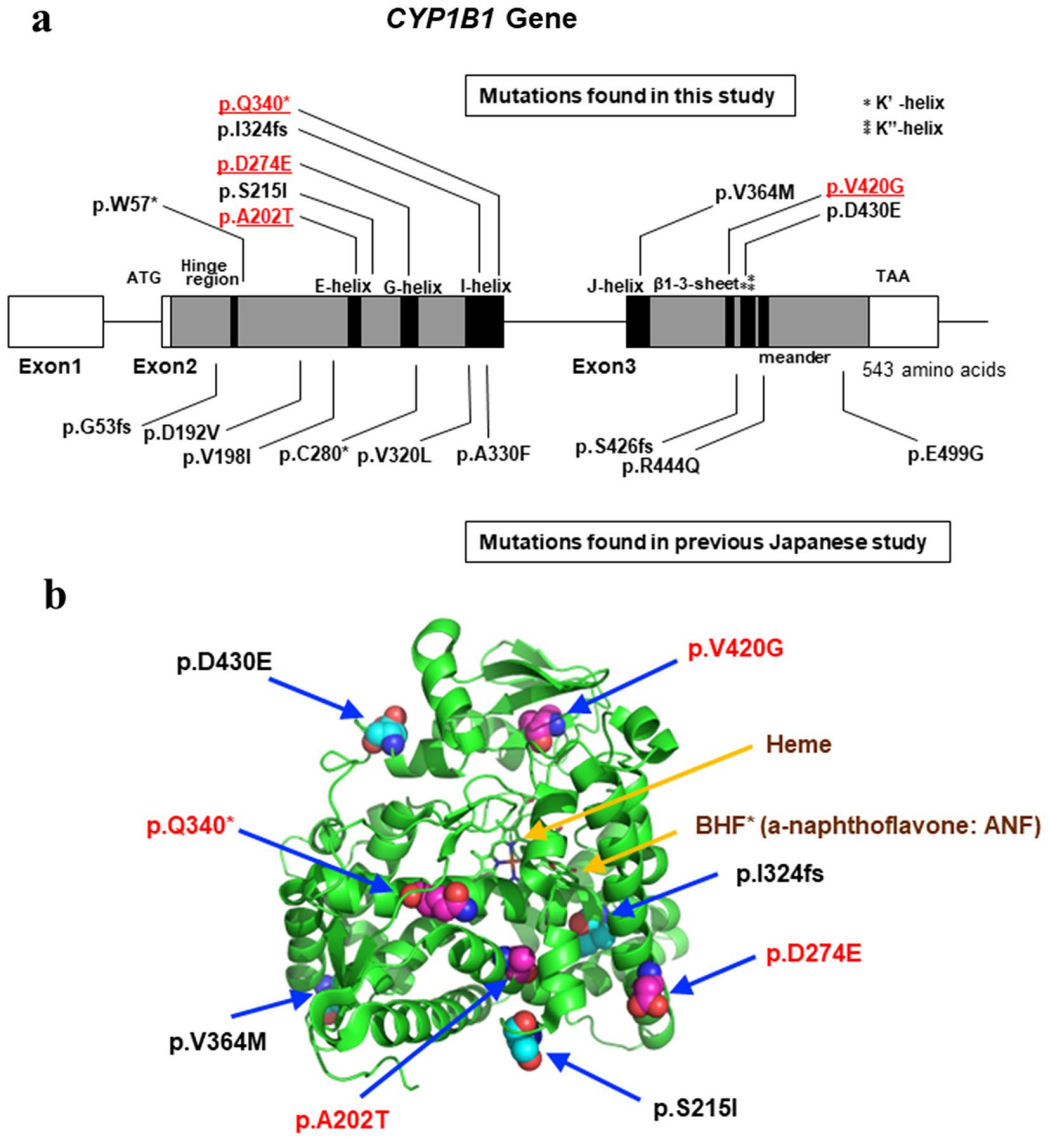
Spectrum of CYP1B1 mutations in Japanese individuals and positions of mutations in the CYP1B1 protein. **a** CYP1B1 mutations found in this study and in a previous Japanese study are shown. The mutations found in this study are displayed above the exons. The p.A202T, p.D274E, p.Q340* and p.V420G mutations were novel in this study and are shown in red and underlined, whereas the p.W57*, p.S215I, p.I324fs, p.V364M and p.D430E mutations were found previously. Mutations found in previous Japanese studies are displayed below the exon. Note that these mutations are found in both exon 2 and exon 3, but the frequency is much greater in exon 2 than in exon 3. **b** Mutations mapped to the CYP1B1 structure. The structure of CYP1B1 in complex with heme and a-naphthoflavone (ANF) is shown. The mutations identified in this study were mapped to the protein structure, and novel mutations identified in this study are shown in red. BHF; 2-phenyl-benzo(h)chromen-4-one, also known as ANF

The *CYP1B1* gene consists of three exons, one noncoding exon (exon 1) and two coding exons (exons 2 and 3). We mapped the mutations found in our sequencing analyses to these exons (Fig. 3a). Six mutations were located in exon 2, i.e., p.W57*, p.A202T, p.S215I, p.D274E, p.1324 fs, and p.Q340*, while three were located in exon 3, i.e., p.V363M, p.V420G, and p.430E. Mutations identified in previous studies in Japan are also shown below the exons (Fig. 3a). There was no hotspot of *CYP1B1* gene mutations, and the mutations were scattered rather widely in exons 2 and 3.

As the structure of the CYP1B1 protein has been elucidated [13], we mapped the mutations identified in this study onto the CYP1B1 protein structure (Fig. 3b). CYP1B1 forms a complex with heme and associates with substrate xenobiotics, such as a-naphthoflavone (ANF). We found that many of the mutations identified in this study localize to the helical structures of the CYP1B1 protein (Fig. 3b), and the results of the analysis revealed that two mutations with recessive inheritance, p.A202T and p.D274E, elicit structural deformities that attenuate CYP1B1 activity. In contrast, three mutations whose heterozygosity appeared to cause CG, i.e., p.V420G, p.S215I and p.D430E, were mapped rather to the surface of the CYP1B1 protein.

### Structural characterization of the CYP1B1 mutations

**Fig. 4:**
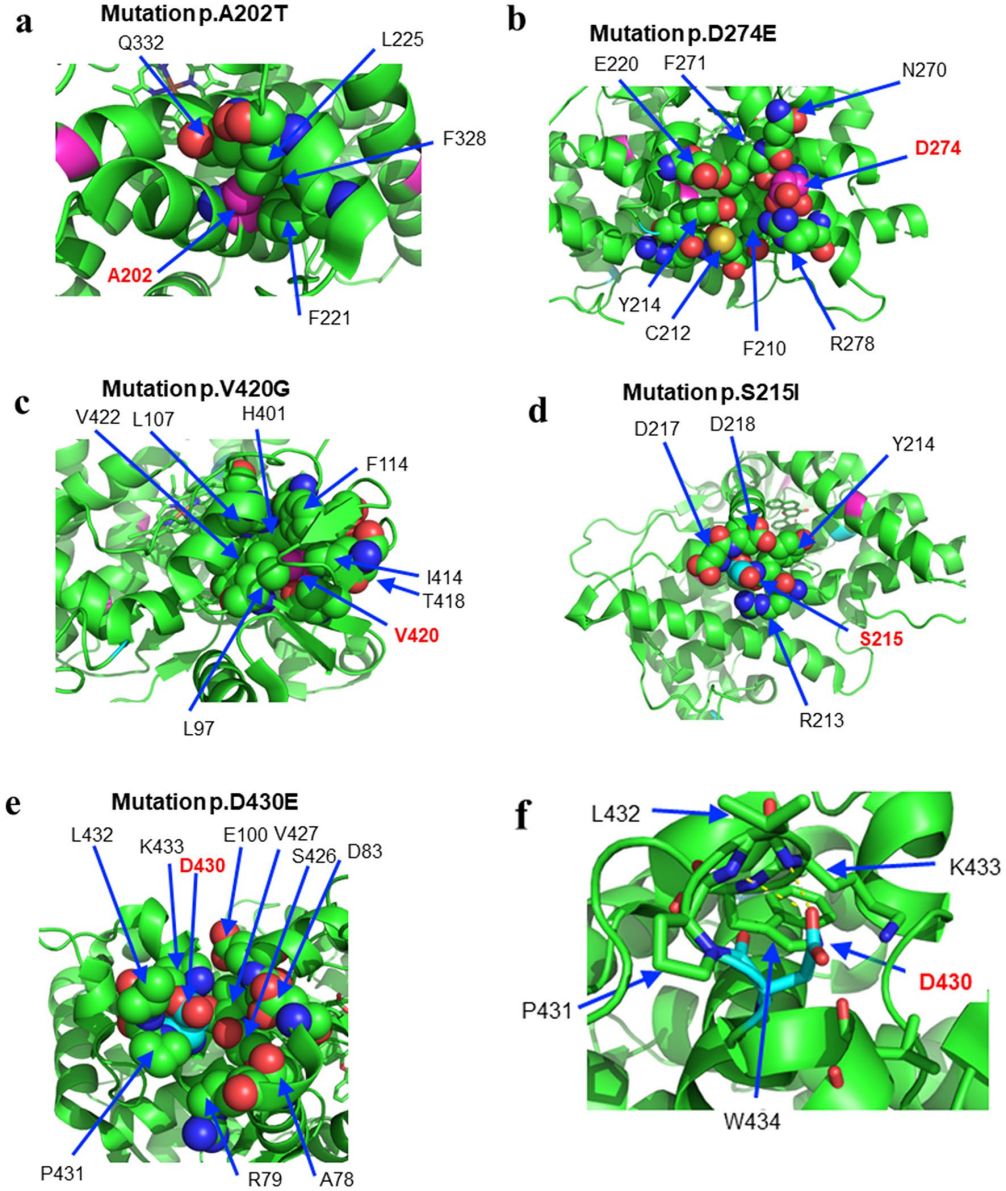
Relationships between each CYP1B1 mutation and the CYP1B1 protein structure. The newly identified mutations were mapped to the CYP1B1 protein structure. **a** p.A202T mutation. The helical structure surrounding Ala is destroyed by the substitution mutation to Thr. **b** p.D274E mutation. Mutation to Glu breaks the interaction of the Asp side chain with the side chain of R278 or makes the interaction unstable. **c** p.V420G mutation. The V420 residue resides on a short β-sheet with side chains interacting with surrounding hydrophobic residues, and the V420G mutation generates a hole in the hydrophobic core. **d** p.S215I mutation. S215 is in the loop between the E-F helices, and the side chain is exposed on the surface, so a hydrophobic molecular surface might be generated. **e** & **f** p.D430E mutation. The D430 residue resides in the loop between K’-K’’ helices, and the side chain of D430 is exposed on the surface. The hydrogen bond formed between the main chain of L432 or K433 and the side chain of D430 might be broken by the mutation to Glu, causing a local structural change

In this study, we found three new nonsynonymous mutations (p.A202T, p.D274E, and p.V420G) and one stop codon mutation (p.Q340*). Therefore, we mapped the CYP1B1 mutations to the CYP1B1 protein structure. Closer examinations revealed that these three substitution mutations provoked structural deformities in the CYP1B1 protein. In the p.A202T substitution mutation in Family 053, the Ala side chain contacts surrounding residues. However, the helical structure surrounding Ala seems to be destroyed by the substitution mutation to Thr (Fig. 4a). Similarly, in the p.D274E mutation (Family 129), the Asp residue resides on the G-helix, with the side chain exposed on the surface (Fig. 4b). The Asp side chain seems to interact with the side chain of R278, while a mutation to Glu breaks the interaction or makes the interaction unstable. Therefore, E274 is located closer to E220 than D274 is (Fig. 4b).

Overall, we surmise that these structural deformities attenuate CYP1B1 activity. These two patients in Family 053 and Family 129 were compound heterozygotes of p.A202T/p.1324 fs and p.D274E/p. Q340*, respectively, suggesting that the recessive inheritance of these mutations caused CG. In contrast, the proband of the p.V420G mutation in Family 020 appeared to be heterozygous for the mutation. The V420 residue resides not on the helix but on a short β-sheet with side chains interacting with surrounding hydrophobic residues (Fig. 4c).

### Other heterozygous mutations of *CYP1B1* in CG patients in this study

Many of the CG patients analyzed thus far were found to harbor *CYP1B1* mutations in a homozygous or compound heterozygous manner, suggesting that mutations in the *CYP1B1* gene adopt a recessive pattern. In contrast, the p.V420G mutation in Family 020, along with the p.S215I and p.D430E substitution mutations in Families 108 and 040, respectively, were found to be heterozygous. In the p.S215I mutation, S215 is in the loop between the E-F helices, the side chain is exposed on the surface, and a hydrophobic molecular surface is generated (Fig. 4d).

Similarly, the D430 residue resides in the loop between the K’-K’’ helices, and the side chain of D430 is exposed on the surface of the molecule (Fig. 4e and f). In addition, the hydrogen bond formed between the main chain of L432 or K433 and the side chain of D430 might be broken by the mutation to glutamic acid, causing a local structural change. Closer investigations of the structure‒function relationships of these mutants await further studies.

### *FOXC1* gene screening

**Fig. 5:**
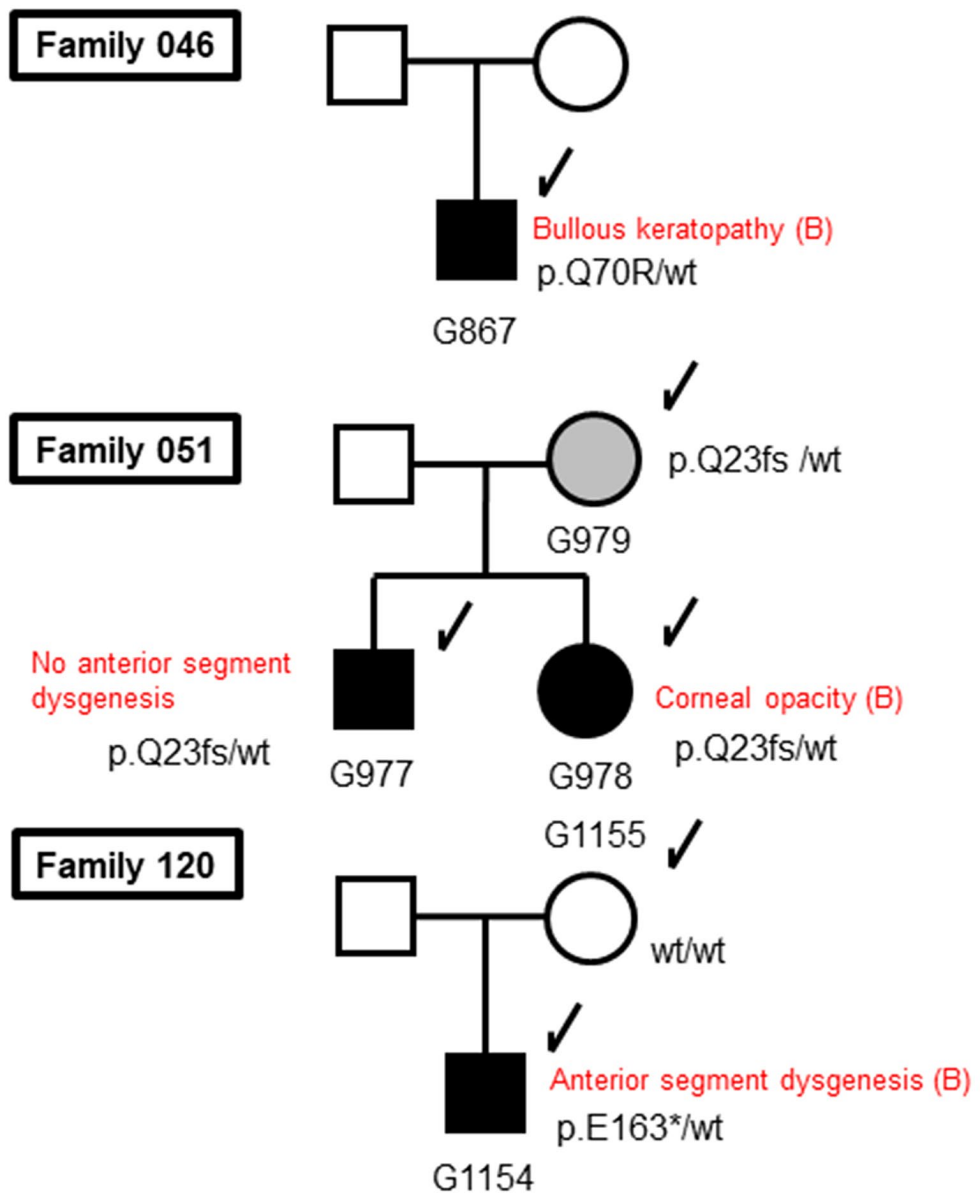
Pedigrees with *FOXC1* mutations. The genotypes are indicated below the symbols. Arrows indicate the tested family members. Closed symbols indicate the subjects with CG phenotypes. A gray symbol indicates a subject with a heterozygous mutation but without the CG phenotype. Open symbols indicate subjects with no CG phenotypes. wt: wild-type allele

In our sequencing analysis of CG patients (see Fig. 1), we identified three mutations in the *FOXC1* gene locus associated with CG families (Fig. 5; Table 3). To our knowledge, these three *FOXC1* gene mutations have not been described to date. The allele frequency of these three substitution mutations was zero in ToMMo 38KJPN, a whole-genome reference panel that covers approximately 38,000 individuals in the Japanese general population [29], and we could not find them in the gnomAD browser (https://gnomad.broadinstitute.org/). These findings indicate that these three mutations are rare.

**Table 3:** *FOXC1* gene: genotype–phenotype correlations with mutations reported in this study

Family & Subject No.	Sex	AA change	Base change^1^	Gene location of mutations	Allele Frequency in ToMMo 38KJPN^2^	Allele Frequency in gnomAD browser^3^	Clin Var	ACMG interpretation	Age at Onset	Age at gene testing	Affected eye	Surgical intervention		Current IOP (mmHg) *R*/L	Visual acuity *R*/L	Clinical phenotype
												*R*	L			
051 G977	M	p.Q23fs	c.67delC	AD-1	0	NA	NA	PVS1, PS4, PM1,2	FMB	22	Bilateral	1x TLO	1x TLO	30/27	0.8/1.5	No anterior segment dysgenesis
051 G978	F	p.Q23fs	c.67delC	AD-1	0	NA	NA	PVS1, PS4, PM1,2	FMB	20	Bilateral	1x TLO, 1x TLE	1x TLO, 1x TLE	13/33	0.15/NLP	R) Corneal opacity, mitral regurgitation
046 G867	M	p.Q70R	c.A209G	Forkhead Domain	0	NA	NA	PS4,PM1,2,6	FMB	63	Bilateral	Multiple TLO	Multiple TLO	12/22	0.01/LP	B) Bullous Keratopathy
120 G1154	M	p.E163*	c.G487T	Forkhead Domain	0	NA	Pathogenic	PVS1, PS4, PM1,2	FMB	18	Bilateral	1x TLO, 1 x tube shunt	1x TLO, 2 x tube shunt	12/16	CF/LP	B) Anterior segment dysgenesis, aortic regurgitation

(1) Reference sequence NM_001453. (2) The allele frequency of the reference was derived from the 38KJPN database distributed by the Tohoku Medical Megabank Organization

M: Male, F: Female, NA: Not applicable, FMB: First few months after birth, TLO: Trabeculotomy, TLE: Trabeculectomy, NLP: No light perception, LP: Light perception CF: Counting fingers

**Fig. 6:**
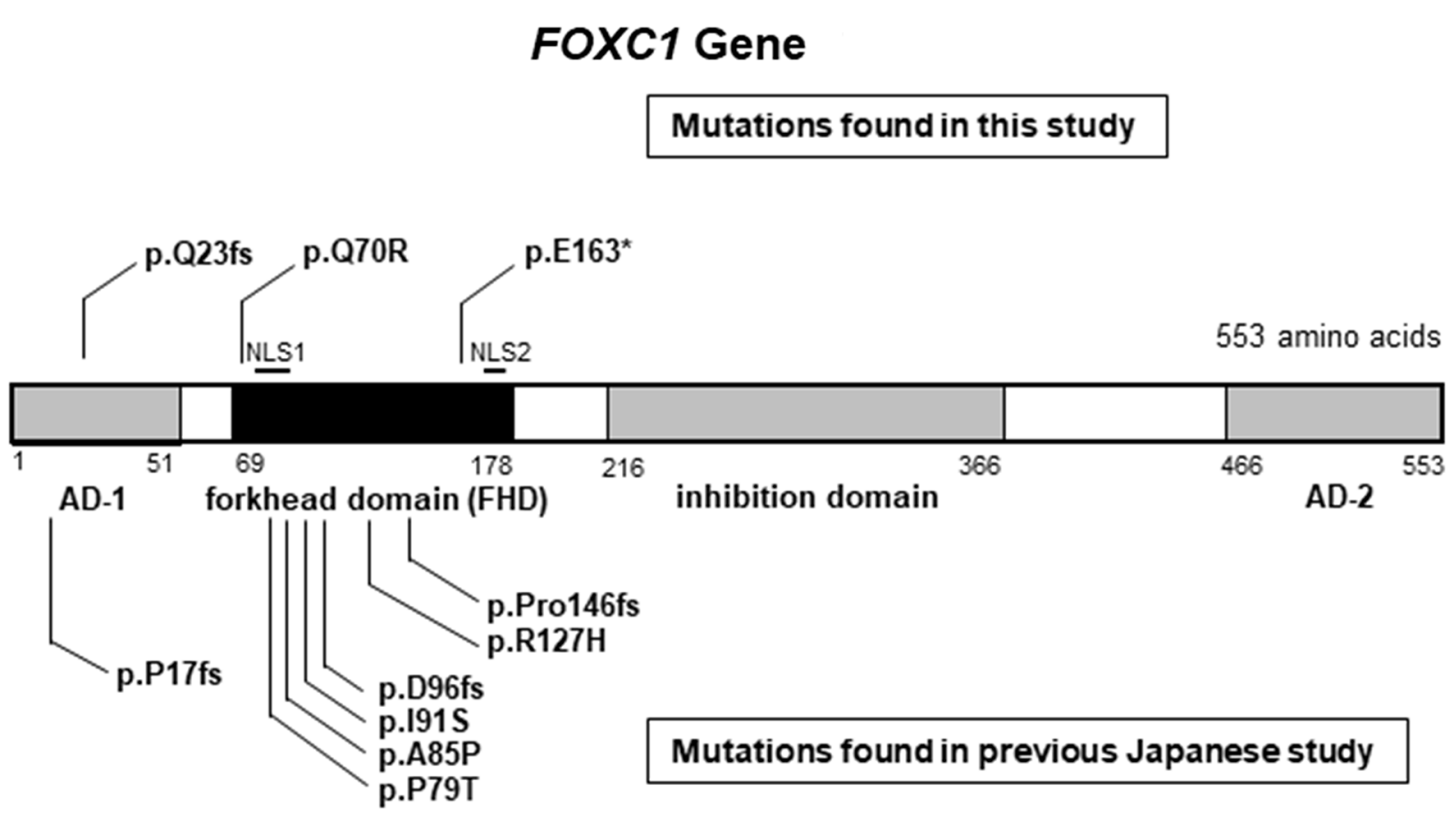
Spectrum of FOXC1 mutations in Japanese individuals. FOXC1 mutations found in this study and mutations found in a previous Japanese study are shown. The mutations found in this study are displayed above the exons. All three mutations were novel (identified in this study). Mutations found in a previous Japanese study are displayed below the exon. All the mutations exist either in the AD-1 domain or in the FHD

A novel frameshift mutation (c.67delC: p.Q23fs) in Family 051 was found to generate a truncated 43-amino acid FOXC1 protein instead of the 553 full-length amino acid sequence (Fig. 6). This truncated protein lacks the forkhead domain (FHD) (residues 69 to 178) and includes two lesions in nuclear localization signal 1 (NLS1; from residues 77 to 93) and nuclear localization signal 2 (NLS2; from residues 169 to 176) of FOXC1 [35]. The p.Q23fs mutation was found in a brother and sister of Family 051 (Fig. 5). Mutations in the *FOXC1* gene are reportedly transmitted in an autosomal dominant manner [19]. Intriguingly, the p.Q23fs mutation was also found in the mother (Fig. 5). Although the mother did not suffer from overt glaucoma, upon closer examination she did show high insertion of the iris at the anterior chamber angle. One of the probands (G978) in Family 051 later suffered from mitral valve regurgitation (Fig. 5).

All the patients with *FOXC1* mutations were bilaterally affected, with early disease onset occurring within 4 months of age, and underwent surgical procedures in both eyes (Table 3). In addition to the p.Q23fs mutation (Family 051), two additional *FOXC1* mutations, p.Q70R (Family 046) and p.E163* (Family 120), were found within the FHD (Fig. 6) and are in the same domains as those found in a previous Japanese study [36, 37]. The patient with the p.E163* nonsense mutation in Family 120 was found to suffer from anterior segment dysgenesis and aortic valve regurgitation.

## Discussion

We analyzed 29 CG families and identified 12 families with causative variants in either the *CYP1B1* or *FOXC1* gene. All individuals with *CYP1B1* or *FOXC1* mutations were severely affected by infantile-onset CG. Of the 9 families with *CYP1B1* mutations, 5 harbored homozygotic or compound heterozygotic *CYP1B1* mutations, while the remaining 4 appeared to suffer from CG under heterozygotic conditions, suggesting the complicated influence of the *CYP1B1* mutations in CG. We also identified 3 novel mutations in the *FOXC1* gene, all of which provoke CG in an autosomal dominant manner. Based on these results, we propose that panel sequencing of the *CYP1B1* and *FOXC1* genes will be useful for the diagnosis of the CG in Japanese individuals.

The incidence of *CYP1B1* gene mutations in this study (9/29; 31%) is comparable to that reported in a Caucasian cohort [38, 39]. We found that 4 families suffered from CG under heterozygous conditions. Consistent with these findings, Mashima and colleagues also found heterozygotic *CYP1B1* mutations in multiple CG patients [16]. These cases are difficult to reconcile with the current notion that *CYP1B1* mutations follow a recessive manner of inheritance. Our protein structure analyses suggest that the mutation site is not decisive for the difference. One plausible hypothesis to explain this situation is to assume that there may be mutations in the gene regulatory regions that affect *CYP1B1* gene expression in a compound heterozygotic manner with nonsynonymous mutations. In support of this hypothesis, homozygous *CYP1B1* knockout mice exhibit developmental abnormalities partially mimicking those in CG, including progressive loss of trabecular meshwork collagen resulting in atrophy of the meshwork and increased IOP [14, 40, 41]. However, the phenotypes of these homozygous knockout mice are rather mild in relation to glaucoma, indicating that these *Cyp1b1*-knockout model mice are not suitable for studying the heterozygous phenotype.

There are ethnic differences in the spectrum of *CYP1B1* mutations. While five cases in four families of the p.V364M mutation were found in this study and in a previous study in Japan [16], this mutation is seldom found in other ethnicities, suggesting the presence of a relatively common founder of the CG-causing mutation in the Japanese population. As numerous CG-related mutations in the *CYP1B1* gene have been reported, a correlation between mutation genotype and disease phenotype is of interest. In this study, we mapped the CYP1B1 mutations to the CYP1B1 protein structure. For p.V420G, we surmise that destabilization of the CYP1B1 structure via mutation of Val at 420 to Gly generates a hole in the hydrophobic core, affecting CYP1B1 activity. For p.S215I, the possibility of structural disruption is rather low. However, because of the substitution of a serine residue with an isoleucine residue, a hydrophobic molecular surface might be generated, which may destabilize CYP1B1 function. Although the molecular basis by which heterozygotes of p.S215I and p.D430E develop CG remains to be determined, these structural analyses (p.S215I and p.D430E) of the CYP1B1 mutations suggest that in heterozygous CG patients, CYP1B1 protein dysfunction per se may not cause distinct contributions to CG pathology according to this structural analysis. Closer investigations of the structure‒function relationships of these mutants await further studies.

Various mutations in the *FOXC1* gene have been implicated in the pathogenesis of a spectrum of ocular disorders [19, 20, 42, 43]. The *FOXC1* gene dosage has been suggested to cause anterior chamber defects [44]. Moreover, mutations within FHD may cause changes in the expression patterns of many genes [45, 46]. In our subjects, clinical ocular features of ARA, such as iris hypoplasia, corectopia, and a prominent anteriorly displaced Schwalbe line (posterior embryotoxon), were neither observed nor subtle (Family120: G1154). These findings suggest that the three mutations identified in this study may affect the migration and/or differentiation of mesenchymal cells that contribute to the anterior segment of the eye [47].

It is reported that various types of *FOXC1* mutations cause various types of anterior chamber dysgenesis in ARA patients with or without systemic features [44]. Unlike glaucoma associated with *MYOC* mutations, i.e., juvenile primary open-angle glaucoma, which has a normal outflow route at birth [48], the *FOXC1* gene mutations identified in this study caused severe angle abnormalities, resulting in perinatal onset of glaucoma. However, the mother (Family 051: G979), who had the p.Q23fs mutation and had a high degree of insertion into the iris at the anterior chamber angle, did not suffer from overt glaucoma (Fig. 5). Further studies are needed to determine the relationship between the extent of angle abnormalities and the variety of *FOXC1* mutations.

The causes of CG in most patients in this study, i.e., 17 out of 29 families, remain unknown. We surmise that there may be heterogeneities in the genetic background of CG. It is reported that mutations in the *CYP1B1* gene occur concomitantly with those in the *MYOC* gene [49]. In this study, we could not find subjects with *MYOC* mutations, implying that the overlap of *CYP1B1* and *MYOC* mutations in CG is not common in the Japanese population. Similarly, it is reported that mutations in the *PXDN* gene cause corneal opacity and CG [23, 24] and that mutations in the *TEK* gene likely underlie CG [9]. Therefore, we are planning to expand the scale of the analysis toward whole-genome sequences and explore new causative genes with strong potential for CG in future studies.

In conclusion, in this study, we screened the *CYP1B1* and *FOXC1* genes and found that *CYP1B1* and *FOXC1* are two major causative genes of CG in Japanese individuals, indicating that panel sequencing of *CYP1B1* and *FOXC1* will be useful for the diagnosis of CG in Japanese individuals.

## Electronic supplementary material

Below is the link to the electronic supplementary material.

Supplementary Material 1

Supplementary Material 2
