# Supplementary material for: Mutations of CYP1B1 and FOXC1 genes for childhood glaucoma in Japanese individuals
Source: Jpn J Ophthalmol. 2024 Aug 19;68(6):688–701. doi: 10.1007/s10384-024-01103-0 (PMC11607050; doi:10.1007/s10384-024-01103-0)
Supplement: Supplementary file 1 — Supplementary Material 1 [file 10384_2024_1103_MOESM1_ESM.docx]

**Supplementary Table 1. Primer sequences for Sanger Sequencing**

| Gene | Primer Name | Exon | Sequence | Annealing temperature |
| --- | --- | --- | --- | --- |
| CYP1B1 | CYP1B1_Ex2-1F | 2 | GCCATTTCTCCAGAGAGTCA | 64 |
|  | CYP1B1_Ex2-1R | 2 | AACTCTTCGTTGTGGCTGAG |  |
|  | CYP1B1_Ex2-2F | 2 | ATGATGCGCAACTTCTTCAC | 65 |
|  | CYP1B1_Ex2-2R | 2 | CTGCTTGCAAACTCAGCATA |  |
|  | CYP1B1_Ex3-1F | 3 | TGCAAGGCCTATTACAGGAAA | 62 |
|  | CYP1B1_Ex3-1R | 3 | AGAAAGTTCTTCGCCAATGC |  |
|  | CYP1B1_Ex3-2F | 3 | CACTATTCCTCATGCCACCA | 65 |
|  | CYP1B1_Ex3-2R | 3 | GCAGCACAAAAGAGGAACTG |  |
| FOXC1 | FOXC1_af | 1 | AACTCCCTGGGAGTGGTGCCCTA | 68  68  68 |
|  | FOXC1_ar | 1 | CGGCTCCTTGAGGTGCAGCCT |  |
|  | FOXC1_bf | 1 | GGCGCTTCAAGAAGAAGGACGC |  |
|  | FOXC1_br | 1 | GCAGCGACGTCATGATGTTGTCCA |  |
|  | FOXC1_cf | 1 | TCAGCCTGGACGGTGCGGATT |  |
|  | FOXC1_cr | 1 | TGGTGACCGGAGGCAGAGAGTAG |  |
