# Supplementary material for: Mutations of CYP1B1 and FOXC1 genes for childhood glaucoma in Japanese individuals
Source: Jpn J Ophthalmol. 2024 Aug 19;68(6):688–701. doi: 10.1007/s10384-024-01103-0 (PMC11607050; doi:10.1007/s10384-024-01103-0)
Supplement: Supplementary file 2 — Supplementary Material 2 [file 10384_2024_1103_MOESM2_ESM.docx]

**Supplementary Table 2.**

**Background of the patients recruited in this study**

| Subject No. | Sex | Age at Onset | | Affected Eye | Surgical  procedure | Clinical Phenotype | |
| --- | --- | --- | --- | --- | --- | --- | --- |
|  |  |  |  |  |  |  |  |
| G072 | F | | FMB | Bilateral | + | B) Iris stromal hypoplasia with loss of crypts | |
| G267  G279* | F  M | | FMB  FMB | Bilateral  Right | +  ++ | B) Corneal opacity  R) High insertion | |
| G320  G461  G493*  G551*  G552* | M  M  F  M | | FMB  FMB  FMB  FMB  FMB | Bilateral  Bilateral  Bilateral  Bilateral  Bilateral | ++  +  ++  ++  + | B) Corneal opacity  B) Corneal enlargement (>13 mm)  R) Corneal opacity, B) Haab’s striae  B) Iris stromal hypoplasia with loss of crypts  B) Iris stromal hypoplasia with loss of crypts | |
| G538  G558*  G588 | M  M  M | | FMB  FMB  FMB | Bilateral  Bilateral  Bilateral | +  ++  + | B) Corneal opacity, L) Retinal detachment  B) Corneal enlargement (>13 mm), Haab’s striae, R) Corneal opacity  B) Corneal enlargement, Haab’s striae, Corneal opacity | |
| G616  G773  G867* | M  F  M | | FMB  FMB (1month)  FMB | Bilateral  Bilateral  Bilateral | +  ++  ++ | B) Iris stromal hypoplasia with loss of crypts  B) Anterior segment dysgenesis, high insertion of the iris  B) Bullous Keratopathy | |
| G881  G977*  G978* | F  M  F | | FMB  FMB  FMB | Bilateral  Bilateral  Bilateral | +  +  ++ | B) Elevated IOP associated with corneal edema  No anterior segment dysgenesis  R) Corneal opacity, mitral regurgitation | |
| G985  G987*  G1026  G1027  G1028  G1029 | F  M  M  M  M | | 6 months  FMB  FMB  FMB  FMB  FMB | Bilateral  Right  Bilateral  Bilateral  Bilateral  Bilateral | +  +  +  +  +  + | B) Prominent Haab's striae  R) Extreme high IOP  B) Elevated IOP associated with corneal edema  B) Elevated IOP associated with corneal edema  B) Corneal opacity  B) Elevated IOP associated with corneal edema | |
| G1149  G1150*  G1152*  G1154*  G1157  G1178  G1199*  G1207* | F  M  M  M  M  M  M  M | | FMB  FMB  FMB  FMB  FMB  FMB  1.y.o  FMB | Bilateral  Bilateral  Bilateral  Bilateral  Bilateral  Bilateral  Bilateral  Bilateral | +  ++  ++  ++  +  +  ++  ++ | B) Elevated IOP associated with corneal edema  B) Anterior segment dysgenesis, high insertion of the iris  B) Peters' anomaly, anterior segment dysgenesis, corneal opacity, high insertion of the iris  B) Anterior segment dysgenesis, aortic regurgitation  B) Elevated IOP associated with corneal edema  B) Elevated IOP associated with corneal edema  B) Peters' anomaly, anterior segment dysgenesis, corectopia, high insertion of the iris  B) High insertion, anterior segment dysgenesis, corneal opacity |  |

* 14 cases in which mutations were detected in this study. M: male, F: female, FMB: ﬁrst few months after birth.

For surgical procedure, one plus sign indicates a single surgery, and two plus signs indicate multiple surgeries.

We studied 31 Japanese patients with CG younger than 3 years of age from 29 families, which became prevalent before 3 years of age. Each family had a pedigree pattern with suspected recessive inheritance or a sporadic pattern. Parental consanguinity was not present in any of the patients.
